# Prolonged pediatric intensive care unit (PICU) admission, challenges in diagnosis and treatment in a child with hyper IgM syndrome in a tertiary hospital in Tanzania: a case report

**DOI:** 10.11604/pamj.2024.49.33.42418

**Published:** 2024-10-11

**Authors:** Aika Shoo, Evance Godfrey, Deogratius Mally, Yasser Said, Mary Dealmeida, Kandi Muze, Namala Mkopi

**Affiliations:** 1 Muhimbili National Hospital, Dar es Salaam, Tanzania,; 2 Emory University School of Medicine, Atlanta, Georgia, United States of America

**Keywords:** Hyper immunoglobulin M syndromes, severe sepsis, pediatric intensive care unit, case report

## Abstract

Hyper immunoglobulin M (IgM) syndromes are a collection of uncommon primary combined immunodeficiency disorders. They are characterized by recurrent bacterial infections due to low levels of IgG, IgA, and IgE, while IgM levels remain normal or high. These conditions stem from a mutation in the CD40 ligand gene or disruptions in the CD40-signaling pathway. Those affected face increased susceptibility to frequent bacterial infections, an elevated likelihood of autoimmune issues, and early-onset malignancies. These syndromes are rare and account for a small fraction of immunodeficiency cases. We describe a case of an African infant, who had a prolonged pediatric intensive care unit admission due to recurrent and severe infections which took a prolonged course of medication to be treated. After a diagnostic workup, a diagnosis of X-linked hyper IgM syndrome was established, and currently, our child is on monthly replacement of IV immunoglobulin and daily prophylactic cotrimoxazole tablets. Early diagnosis of primary immunodeficiency disorders reduces the incidence of infections and the severity of complications. This case demonstrates the consequences of delayed diagnosis and resulting in a prolonged hospital stay.

## Introduction

Hyper Immunoglobulin M syndromes (HIGM) are a heterogenous group of primary immunodeficiency disorder characterized by production of normal or increased levels of serum immunoglobulin M (IgM) and insufficient quantities of immunoglobulin G (IgG), immunoglobulin A (IgA) and immunoglobulin E (IgE). This is associated with poor antibody function. The prevalence of X-linked HIGM varies in different ethnicities in the world. The approximated incidence is 1 in 1,030,000 live births [1,2]. In a normal immune response to a new antigen, B cells initially produce IgM antibodies, then transition to synthesizing IgG, IgA, and IgE antibodies, enhancing the protection of tissues and mucosal surfaces. Hyper immunoglobulin M syndrome (HIGM) can emerge as either an acquired or hereditary condition, with either X-linked or autosomal patterns of inheritance. All variations of this syndrome are characterized by a defective class-switch recombination (CSR) process [3]. Hyper immunoglobulin M syndrome is a consequence of mutations in the CD40 ligand (CD40L) gene, also known as tumour necrosis factor superfamily 5 genes (TNFSF5) situated on the X chromosome at q26. This gene encodes CD40L expressed on antigen-presenting cells including B cells, dendritic cells, and macrophages, crucial for the immune response. When mutated, the production of CD40 antigen ligand is impaired, leading to the inability of B cells to receive signals from T cells leading to impaired class switching. As a result, B cells cannot transition their antibody production to IgA, IgG, and IgE, leading to decreased levels of IgG, IgA and IgE but normal to elevated levels of IgM in the serum [4].

Patients with hyper IgM are at significant risk for recurrent infections. Most commonly, it involves sino-pulmonary infections by encapsulated organisms such as *Streptococcus pneumoniae*. Opportunistic infections including *Cryptosporidium* can present as chronic or protracted diarrhea. Other pathogens including *Pneumocystis* and *Histoplasma* can result in failure to thrive. There is a potential for acquiring central nervous system (CNS) toxoplasmosis and cryptococcal meningoencephalitis. Studies have also indicated an increased likelihood of developing autoimmune diseases and even malignancies later in life. Some individuals with hyper IgM also present with recurring neutropenia [1,5]. Presented here is a case of an infant from Tanzania harboring a novel mutation in the CD40 ligand gene. This child required a three-month stay in our PICU due to frequent and recurring infections, coupled with a delay in diagnosing the condition. To the best of our knowledge, this represents the first documented case within our country.

## Patient and observation

**Patient information:** a five-month-old male infant was admitted to our PICU due to respiratory failure attributed to severe pneumonia. The patient had a two-month history of recurring fever, cough, and chronic diarrhea, which had led to significant failure to thrive. Prior to his PICU admission, he had undergone multiple antibiotic courses and had even been initiated on anti-tuberculosis drugs at one point, without experiencing relief from his symptoms.

**Timeline of the current episode:** as his respiratory distress escalated significantly, he was referred to our PICU for specialized respiratory support. The infant was born at term, weighing 3 kg, following an uneventful pregnancy. His parents were of non-consanguineous African descent. He is the sole child in the family, having lost an older sibling at one year of age within our facility due to similar complaints, though genetic testing had not been conducted at that time. On his admission, he was also diagnosed with severe pneumonia with respiratory failure.

**Clinical findings:** upon admission, the baby presented as lethargic, markedly undernourished, and weighing 3 kg. He displayed palmar pallor, pronounced oral pharyngeal thrush, and evident signs of severe respiratory distress marked by bilateral coarse crepitations and rhonchi.

**Figure 1:**
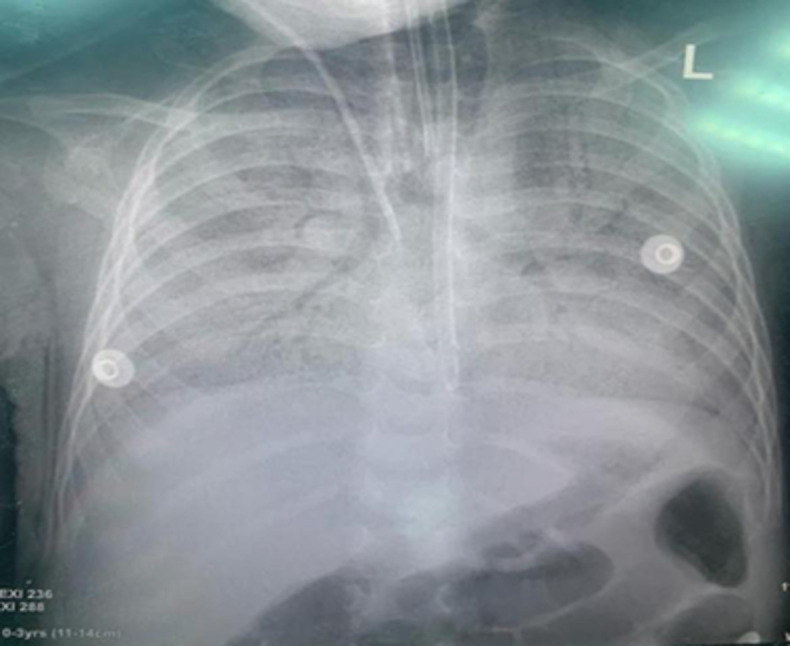
chest X-ray showing bilateral consolidation

**Diagnostic assessment:** an initial complete blood count revealed leukocytosis with neutrophilia. Arterial blood gas analysis indicated severe uncompensated metabolic acidosis. Elevated C-reactive protein levels were observed. Chest X-ray displayed bilateral consolidation, as illustrated in (Figure 1). Notably, HIV serology, gene expert testing for tuberculosis, and COVID-19 serology all returned negative results. Liver and renal profiles were within normal ranges. The first blood culture yielded the growth of *Escherichia coli*, leading to the initiation of a multifaceted antibiotic regimen. Subsequent blood culture, conducted a month later, detected *Staphylococcus aureus* growth. Unfortunately, the patient's condition worsened despite these antimicrobial regimens (Table 1).

**Table 1:** summary of clinical presentation, pathogen isolated and treatment provided

Age	Clinical presentation	Pathogen	Treatment
5 months	Fever, oral thrush, difficulty in breathing	Swab culture-candida; blood culture-*E. coli*	Fluconazole; meropenem
6 months 2 weeks	Fever, difficulty in breathing	Blood culture-*S. aureus*	Meropenem
7 months	Fever, difficulty in breathing	Blood culture-pseudomonas aeruginosa	Tazobactam /piperacillin and amikacin
7 months and 2 weeks	Fever, vomiting	Blood smear-plasmodium falciparum	Artesunate
8 months	Fever, difficulty in breathing	Blood culture-*S. aureus*	Ciprofloxacin
9 months	Fever, skin abscesses	Swab culture-*S. aureus*	Clindamycin

Serial immunoglobulin assessments demonstrated normal serum IgM levels at 0.69 g/L and serum IgG levels, ranging between 3.1 g/L and 3.72 g/L. Serum IgA levels remained constantly low between 0.25 g/L and 0.47 g/L. Considering these findings, genetic studies were pursued, ultimately revealing a mutation in the CD40 ligand gene, specifically NM_000074.2: c.347-1G>A variant. This confirmed the diagnosis of hyper IgM-type 1 (HIGM1) after two and half months of his stay in PICU.

**Therapeutic intervention:** the patient had been intubated and mechanically ventilated for two months, due to severe acute respiratory distress. He completed a course of IV meropenem due to an initial blood culture which grew *E. coli*. Throughout his PICU stay, he developed recurrent infections and received multiple subsequent antimicrobial courses (Table 1).

**Follow-up and outcome of intervention:** he remained in the PICU for a total of three months, following which, he was transitioned to the pediatric ward and remained there for an additional month prior to being discharged home. Following the confirmation of his diagnosis, a regimen of monthly IV immunoglobulins (IVIG) injections was initiated and prescribed a daily dose of cotrimoxazole (240 mg) as prophylactic treatment. Subsequent to the initiation of these interventions, the frequency of recurrent infections has notably decreased. His weight has improved and he has experienced positive developmental growth.

**Patient perspective:** parents were happy that the child improved; however, they were sad that he will need prolonged and expensive treatment with immunoglobin and antibiotics prophylaxis.

**Informed consent:** written informed consent to publish this case was obtained from the parents.

## Discussion

Hyper IgM syndromes are a group of primary immunodeficiency disorders caused by defects in the CD40 ligand or CD40-signaling pathway, leading to impaired B-cell proliferation, immunoglobulin class switch recombination (CSR), and germinal center formation [4]. As a consequence, children with hyper IgM syndrome have decreased concentrations of serum IgG, IgA, and IgE and normal or elevated levels of IgM, leading to increased susceptibility to recurrent and severe infections resulting in failure to thrive and recurrent hospital admissions. Some can even develop arthritis, malignancy, and autoimmune diseases later in life. The most common form is the X-linked type, due to mutation of the CD40 ligand, which accounts for about 70% of individuals with HIGM syndromes [6].

As described above, our child presented with low levels of IgA and normal levels of IgM and IgG. Having normal IgG levels at 5 months of age, could be affected by maternal IgG. Gene sequencing analysis revealed an NM_000074.2: c.347-1G>A, a hemizygous variant of the CD40L gene. This variant has been previously described as a disease-causing for hyper IgM syndrome [7]. Diagnosis and management of primary immunodeficiency diseases in sub-Saharan Africa remains a persistent challenge. Most patients with HIGM syndrome develop clinical symptoms during their first or second year of life, but some children can exhibit symptoms as early as six months of age due to the decline in maternal antibody titers. Some cases might even manifest as early as three months of age [4,7] as for the case of our child. He started presenting with symptoms of recurrent illness at three months of age. By five months of age, he was admitted to PICU, where he required mechanical ventilation and management for severe sepsis. He incurred complications of prolonged intensive care unit (ICU) stay, including ventilator-associated pneumonia, pressure sores, and critical illness myopathy, which all required multidisciplinary care.

In this case, there was a delay in reaching diagnosis, for the genetic evaluation, blood samples were taken abroad, which further took time to get results back. This also contributed to delay in diagnosis, prolonged PICU stay, and increased risk of acquiring nosocomial infections as shown in Table 1. Despite the encountered challenges, vigilant monitoring and prompt septic workup contributed to the patient's eventual recovery and subsequent discharge from the PICU. Treatment options for children with HIGM syndrome consist of monthly intravenous immunoglobulin (IVIG) replacement of the missing IgG antibodies, and daily prophylaxis dose of Co-trimoxazole to prevent against opportunistic infections. Additionally, timely infection treatment is essential. Upon confirmation of the diagnosis, our management involved the administration of monthly IVIG injections and a daily dose of co-trimoxazole syrup, which continues to the present day. The importance of maintaining hygiene, including water boiling, was emphasized to parents to prevent recurrent cryptosporidium infection [8]. The definitive cornerstone of treatment resides in hematopoietic stem cell transplantation, and those with advanced liver disease might also benefit from liver transplantation [9,10].

## Conclusion

Our case report underscores the critical significance of early and timely diagnosis. In our patient's situation, the diagnostic delay led to increased morbidity. Research has indicated that despite optimal medical intervention, the prognosis remains unfavorable, with fewer individuals surviving beyond their third decade of life due to heightened risks of autoimmune diseases and malignancies.
